# Evaluation of outcomes for psychosis and epilepsy treatment delivered by primary health care workers in Nepal: a cohort study

**DOI:** 10.1186/s13033-017-0177-8

**Published:** 2017-11-25

**Authors:** M. J. D. Jordans, L. Aldridge, N. P. Luitel, F. Baingana, B. A. Kohrt

**Affiliations:** 1 Centre for Global Mental Health, Institute of Psychiatry, Psychology and Neuroscience, King’s College London, London, UK; 2 Research and Development Department, HealthNet TPO, Amsterdam, Netherlands; 3 Research Department, Transcultural Psychosocial Organization–Nepal (TPO), Kathmandu, Nepal; 4 School of Public Health, Makerere University, Kampala, Uganda; 5 Department of Psychiatry, George Washington University, Washington, DC USA

**Keywords:** Evaluation, Community mental health, Primary health care, Psychosis, Epilepsy, low and middle income country, Nepal

## Abstract

**Background:**

Most evaluations of task-shifting have focused on common mental disorders. Much less work has been done on severe mental neurological and substance use (MNS) disorders, such as chronic psychosis and epilepsy. Given the high burden associated with severe MNS and the lack of mental health professionals in low and middle income countries, evaluations on the impact of task-shifting for these disorders are important.

**Methods:**

In a rural district of Nepal, a community mental health program, based on World Health Organization’s Mental Health Gap Action Programme guidelines, was evaluated using a cohort study design. People with epilepsy and psychotic disorders were interviewed at treatment initiation and at 12-month follow-up. We also compared a group that was offered a comprehensive package of care (medication combined with psychosocial interventions, such as counselling and peer support groups) to a group that received medication only.

**Results:**

One-hundred nineteen persons were enrolled in the epilepsy cohort (EC) and 85 in the psychosis cohort (PC). The patients were enrolled in either the comprehensive package (n = 157) or medication only (n = 47). There was significant improvement (P < 0.0001) in psychosis symptoms (PC: Z = 6.78, *r* = 0.80) and depressive symptoms (EC: Z = 7.43, r = 0.73; PC: Z = 6.02, *r* = 0.70), seizures (EC: Z = 6.78), functional disability (EC: Z = 6.38, *r* = 0.67; PC: Z = 4.60, *r* = 0.57), family and caregiver burden (EC: Z = 8.09, r = 0.85; PC: Z = 6.81, r = 0.84), and social behaviour (PC: Z = 5.94, *r* = 0.84). There was greater risk reduction for recent seizures among people with epilepsy in the comprehensive treatment package vs. medication only (risk ratio = 0.52, 95% CI 0.29–0.95; P = 0.03); no other significant differences were observed between treatment arms.

**Conclusions:**

A community mental health program in Nepal, implemented by non-specialists, resulted in moderate to large effects among people with epilepsy or psychosis. A comprehensive package of care, including counselling and patient support groups, appears to offer added clinical benefits for patients with epilepsy. For people with psychosis, the basic package of care (i.e., psychotropic medications) performed similar to the comprehensive package, suggesting a less resource-intensive package may offer comparable results.

## Background

In most low- and middle income countries (LMIC) decentralized mental health services often range from inadequate to nonexistent [1], which is particularly concerning in light of the high burden of disease associated with mental illness globally [2]. Prior research has demonstrated high levels of common mental disorders in rural Nepal [3, 4], and a treatment gap (i.e. the percentage of people with mental illness that are not receiving adequate services) between 94.9 and 91.9 for alcohol use disorder and depression, respectively (Luitel et al., under review). Similar data on severe mental, neurological and substance use (MNS) disorders are lacking for Nepal. The integration of mental health services within primary health care has long been advocated as a strategy to reduce the treatment gap, most recently through a set of clinical guidelines for health workers [5].

Consistent with this approach, the Mental Health Beyond Facilities (mhBeF) project aimed to establish a community-based mental health care program designed to reduce the treatment gap and relieve the clinical and social burden of severe MNS disorders for patients and their families. Patients enrolled in mhBeF receive a Mental Health Care Package (MHCP) developed in accordance with the World Health Organization’s (WHO) Mental Health Gap Action Programme (mhGAP; [6]), which relies on non-specialist health workers in primary care centers as the focal point for the detection and treatment of mental disorders. The MHCP integrates both mental health services into primary health care, which pivots from a facility-based to a community-centric model of treatment, and is comprised of (i) strengthening clinical recognition, referral, assessment and management by non-specialist health care workers and (ii) community level activities such as case detection, family counselling and patient support groups (PSGs).

The present study evaluates the outcomes of a treatment package for a cohort of patients with two MNS disorders, either psychosis or epilepsy, in rural Nepal. The primary objective is to evaluate the clinical and functional outcomes of the full cohort after 12 months of treatment. The secondary objective is to assess the added value of a comprehensive package of services over a minimal package of services. Ultimately the study aims to establish an evidence base for the viability of using this treatment model in Nepal and the potential for assimilation into the Nepal healthcare system at scale.

## Methods

### Setting

The mhBeF program in Nepal was implemented in Pyuthan district between 2013 and 2015. Pyuthan is a district located in the mid hills of the Rapti zone in the mid-western development region of Nepal. Nepal ranks 145 of 187 countries on the human development index (HDI) in 2015. The objective of mhBeF was to develop and implement a comprehensive community-based mental health care plan, in accordance with the mhGAP guidelines, for persons with severe mental disorders (psychosis and bipolar disorder) and epilepsy in Liberia, Uganda, and Nepal [7].

### Intervention arms

The study evaluated patients that were offered a comprehensive care package, which included; (a) clinical assessment and psychotropic treatment by primary health care (PHC) workers following the mhGAP Intervention Guide (after receiving a 9-day training); (b) psychosocial support through individual or family counselling and patient support groups (PSGs) by community counsellors (after receiving a 21-day training, in addition to a base training in counselling); and (c) conducting stigma reduction activities and ensuring follow-up care through home-based care by female community health volunteers (after receiving a 3-day training). Supervision of PHC workers by a psychiatrist was an integral component of the program. In addition the program used a newly developed procedure (Community Informant Detection Tool—CIDT) for pro-active case finding by community members to increase demand for mental health services. The CIDT has been developed in Nepal, and evaluated for accuracy [8] and impact on increased help seeking [9]. One group of the study participants was offered this comprehensive package of care, while a comparison group was offered a more basic set of services consisting only psychotropic medicines (enhanced Treatment As Usual, eTAU) by mhGAP trained health workers, to allow the assessment of the added value of a comprehensive package of services over a minimal package of services. While the program planned to cater for people with psychosis, epilepsy and bipolar disorder, during the implementation phase only 6 people with bipolar were identified and treated. These people have been excluded from the study. Allocation to treatment or control arms was done by health facilities, which were geographically separated to minimize contamination. In the treatment arm we included the health facilities of 12 Village Development Committees (VDC; the smallest administrative unit in Nepal), and health facilities of 5 VDCs were included in the control arm. We first listed all eligible VDCs within the district (excluding those where referral and supervision were not going to be feasible). Next, we separated the health facilities (n = 5) that had received some form of prior mental health training (which was less than the training that was given as part of this study) from those that had never received such training. The former were allocated to the eTAU arm. Among the latter we randomly selected 12 health facilities—the number was set by the maximum capacity for project implementation.

### Sample

Patients with a primary diagnosis of psychosis or epilepsy established by PHC workers trained in mhGAP were recruited into a controlled cohort study. Inclusion into the study followed a three-staged process. First potential respondents were identified as possibly having one of the target disorders (i.e. psychoses and epilepsy) using the CIDT—as described above. Second, all identified individuals were screened using a brief screening instrument developed for the purpose. Third, if a person is found positive on one or more of the target disorders based on the screening procedure, he or she was assessed by trained PHC workers following the mhGAP guidelines for on the target disorders.

### Procedures

All research assistants were selected from Pyuthan district and received a 3-week training. The training included basic communication and interviewing skills, research concepts and ethics, as well supervised practice of administering the surveys. Written consent was sought from all respondents, after a full explanation of the study. All individuals that were diagnosed and provided informed consent, baseline measurement was administered by research assistants. Recruitment took place between June 2014 and May 2015. Ethical approval for this study was obtained from the Nepal Health Research Council (NHRC) (Ref: 874; Reg 173/2013). Adverse events were monitored and responded to following TPO Nepal’s Adverse Events Reporting Procedure.

### Measures

We selected a clinically-appropriate measure to assess symptom reduction for each disorder [1] Psychosis symptoms: people with psychosis completed the positive and negative symptoms scale (PANSS) (10), which has previously been validated in South Asia [11]. For this study we did not use the PANSS general scale, but only the positive and negative symptoms sub-scales (and for analyses combined positive and negative symptoms sub-scales) [2]. Depression symptoms: all participants also completed the Hamilton Depression Scale (HAM-D; [12]), which we included due to the increased risk of depression among people with epilepsy and psychosis [3]. Seizures: For people with epilepsy, a 9-item instrument to measure the number epileptic seizures in the previous 3 months (i.e., recent seizure) was used [13], which was scored dichotomously [4]. Disability: we measured functional disability for all patients through the 12-item version of the WHO Disability Assessment Schedule 2.0 (WHODAS). The WHODAS has been used extensively and validated in many low- and middle-income countries [14], including in Nepal [15]. We used the complex scoring method when calculating patients’ scores, which incorporates item weights [5]. Burden associated with mental illness, an important impact factor for caregivers and families of those with severe disorders, was measured through the burden assessment schedule (BAS; [16]) and two sections of the family interview schedule (FIS; [17]): symptoms and social behavior (FIS-SSB) and impact on caregiver (FIS-IC). The disability and burden measures were administered through family interview conducted by trained research assistants.

Translation of these instruments followed a standardized five-step procedure for translation of instruments for use in transcultural research [18]. The PANSS, HAM-D, and Epilepsy-9 were administered by trained clinicians. The other instruments were administered by trained research assistants.

The primary outcomes for the study were the two disorder-specific clinical outcomes (i.e., PANSS score for psychosis and recent seizure for epilepsy) and associated functional impairment (i.e., WHODAS score). All other domains are considered secondary outcomes. Each measure was administered at baseline and at 12 months follow-up.

### Analysis

We compared baseline characteristics of the study arms using χ^2^ tests for categorical data and an independent samples t-test for the single sample characteristic with continuous data, age of patient. To evaluate each clinical and functional outcome for the full cohort, we conducted a paired T-test for scaled outcomes, which compared baseline and endpoint scores on each measure for all patients. We evaluated the disorder-specific clinical outcome for epilepsy (i.e. recent seizures), using McNemar’s exact χ^2^ for binomial paired data, which compared the proportion of patients with a recent seizure at baseline to that at endpoint. Analyses were carried out on a per protocol basis.

Differences in clinical and functional outcomes across treatment arms were assessed using an analysis of covariance for each outcome. We set the treatment group as a fixed-effect exposure and baseline score as a covariate, with the patient’s score at endpoint as the outcome. For measures that did not meet Levene’s test for equality of variance, we used a logarithmic transformation to account for heteroscedasticity [19]. We compared the reduction in risk of the dichotomous outcome for epilepsy seizure across treatment arms through logistic regression. Again, treatment arm was set as a fixed-effect exposure and recent seizure at baseline as covariate, with recent seizure at endpoint as the outcome.

Upon reviewing the data for the cohort, there were outliers in the distribution of change scores for multiple scaled measures, indicating the possibility of an underlying non-normal distribution of outcomes. We determined post hoc to use the nonparametric equivalent of the paired T-test, the Wilcoxon Signed-Rank Test, when comparing baseline and end point scores for each measure. It was not necessary to select a nonparametric method for analysis between treatment arms despite the presence of outliers since ANCOVA has demonstrated to be robust against violations of non-normality [20].

## Results

Of the 204 patients enrolled at baseline, 85 (42%) and 119 (58%) patients had a primary diagnosis of psychosis and epilepsy, respectively. One hundred fifty-seven (77%) patients were included in the program arm (i.e., comprehensive services package; 63 with psychosis and 94 with epilepsy) and 47 (23%) patients to the control arm (i.e., minimal services package; 22 with psychosis and 25 with epilepsy). The majority of patients were married (64%), Hindu (99%), and did not attend secondary school (72%); approximately half of the patients were male (51%) and worked in agriculture (47%). One hundred seventy-nine (88%) patients completed the clinician interviews at end point, while complete data from family interviews were available for 156 (76%). Table 1 presents the full distribution of sociodemographic characteristics for the sample.

**Table 1:** Sociodemographic characteristics of sample presented by total and subgroup percentages

	Comprehensive treatment package (%)	eTAU (%)	Total (%)	χ^2^ *P* value comparison
	n = 157	n = 47	n = 204	
Disorder				0.42
Psychosis	40	47	42	
Epilepsy	60	53	58	
Male	51	51	51	0.99
Average age^a^	36.5 (14.4)	34.9 (11.5)	36.2 (13.8)	0.49
Highest level of education				
No formal education	38	43	39	
Primary level	32	34	33	
Secondary level	21	19	21	
Higher secondary or above	9	4	8	
Employment category				
Unemployed	22	26	23	
Student or informal	11	43	19	
Agriculture	54	23	47	
Formal or day work	13	9	12	
Caste				0.33
Brahmin/Chhetri	34	23	32	
Dalit	30	32	30	
Janajati/Yogi/Puri/Giri	36	45	38	
Married	61	74	64	0.08
Family income sufficient to sustain yourself? (months per year)				0.26
A little (1–3)	10	17	12	
A quite bit (3–6)	29	34	30	
Very much (6–9)	33	34	33	
Always (9–12)	27	15	25	

^a^Average age presented as values, not percentages; also T-test *p* value presented for age comparison

*eTAU* enhance treatment as usual

The Signed-rank tests provide very strong evidence the cohort made significant improvements in all clinical, functional, and burden-related outcomes. Patients with psychosis made significant improvements in psychotic (Z = 6.78, *r* = 0.80) and depressive (Z = 6.02, *r* = 0.70) symptoms, functional impairment (Z = 4.60, *r* = 0.57), familial burden (BAS: Z = 6.81, r = 0.84; FIS-IC: Z = 5.13, *r* = 0.64), and social behavior (Z = 5.94, *r* = 0.84); all measures *P* < 0.0001. See Tables 2 and 3.

**Table 2:** Clinical outcome summary scores from clinician interview for patients with psychosis

	Program (n = 53)		Control (n = 21)		ANCOVA		Full cohort (n = 74)			
	Mean	SD	Mean	(SD)	Effect size F, partial eta^2^	P-value*	Mean	(SD)	Effect size Z, r	P-value**
PANSS positive and negative					< 0.01, < 0.01	0.96			6.87, 0.80	< 0.0001
Baseline	25.5	12.1	24.9	11.9			25.3	− 12		
end point	7.9	7.4	10.3	12.2			8.6	− 9		
Δ score	− 17.6	12.5	− 14.6	14.1			− 16.8	− 12.9		
PANSS positive					1.22, 0.02	0.22			6.87, 0.80	< 0.0001
Baseline	11.6	6.8	13.1	5.7			12	6.5		
end point	2.7	3.3	3.7	4.6			3	4.6		
Δ score	− 8.9	7.7	− 9.3	7.5			− 9	7.5		
PANSS negative					2.16, 0.03	0.69			6.42, 0.75	< 0.0001
Baseline	13.9	7.3	11.8	7.4			13.3	7.4		
end point	5.2	5.3	6.6	7.7			5.6	6.1		
Δ score	− 8.7	7	− 5.2	8			− 7.7	7.4		
HAM-D					0.02, < 0.01	0.27			6.02, 0.70	< 0.0001
Baseline	15.6	7.2	16.3	7.8			15.8	7.3		
end point	7.7	4.5	8.0	6.7			7.8	6.7		
Δ score	− 7.8	8.0	− 8.4	11.3			− 8.0	9.0		

*HAM-D* Hamilton Depression Scale, *PANSS* positive and negative syndrome scale

* P-value taken from ANCOVA (analysis of covariance) for each scale comparing program vs. control scores at end point, adjusted for baseline score

** P-value from Wilcoxon Signed-Rank Test comparing baseline to end point scores (full cohort)

**Table 3:** Functional and familial burden for psychosis

	Program (n = 48)		Control (n = 17)		ANCOVA		Full cohort T			
	Mean	(SD)	Mean	(SD)	Effect size F, partial eta^2^	P-val*	Mean	(SD)	Effect Size Z, *r*	P-val**
WHODAS					0.52, 0.01	0.52			4.60, 0.57	< 0.0001
Baseline	46.1	(24.2)	37.4	(25.6)			43.8	(24.7)		
end point	23.1	(20.8)	27.1	(30.9)			24.1	(23.7)		
Δ score	− 23.0	(27.0)	− 10.3	(44.3)			− 19.7	(32.6)		
BAS					0.29, < 0.01	0.59			6.81, 0.84	< 0.0001
Baseline	76.5	(8.0)	76.0	(8.2)			76.4	(8.0)		
end point	63.3	(6.5)	62.1	(9.3)			62.9	(7.3)		
Δ score	− 13.3	(8.6)	− 13.9	(11.5)			− 13.4	(9.4)		
FIS: symptoms and social behavior					0.16, < 0.01	0.69			5.94, 0.74	< 0.0001
Baseline	46.5	(9.7)	41.2	(11.4)			45.1	(10.4)		
end point	31.1	(9.4)	31.6	(11.0)			31.2	(9.8)		
Δ Score	− 15.4	(12.3)	− 9.6	(15.8)			− 13.9	(13.4)		
FIS: impact on caregiver					2.02, 0.03	0.16			5.13, 0.64	< 0.0001
Baseline	12.0	(5.9)	10.9	(6.3)			11.7	(6.0)		
end point	5.9	(4.3)	7.6	(5.7)			6.3	(4.7)		
Δ score	− 6.1	(6.4)	− 3.3	(7.9)			− 5.4	(6.9)		

*WHODAS* World Health Organization Disability Assessment Schedule 2.0 Short Version, *BAS* burden assessment schedule, FIS Family Interview Schedule

* P-value taken from ANCOVA (analysis of covariance) for each scale comparing program vs. control scores at end point, adjusted for baseline score

** P-value from Wilcoxon Signed-Rank Test comparing baseline to end point scores (full cohort)

The Chi squared test for paired binomial data indicated very strong evidence for a reduction in the risk of recent seizure at end point compared to baseline for patients with epilepsy (relative risk = 0.33, 95% CI 0.24–0.45; *P* < 0.0001). Patients with epilepsy also demonstrated significant improvements in depressive symptoms (Z = 7.43, r = 0.73), functional impairment (Z = 6.38, r = 0.67), and familial burden (BAS: Z = 8.09, r = 0.85); all measures *P* < 0.0001. See Tables 4 and 5.

**Table 4:** Clinical outcome summary scores from clinician interview for patients with epilepsy

Seizures	Program (n = 83)			Control (n = 22)			Study arm comparison		Full cohort (n = 105)				
	n	%	95% CI	n	%	95% CI	RR (95% CI)	P-value*	n	%	95% CI	RR (95% CI)	P-value**
Recent							0.52 (0.29–0.95)	0.03				0.33 (0.24–0.45)	< 0.0001
Baseline	68	82	(72–90)	20	91	(71–99)			88	84	(75–90)		
end point	19	23	(14–33)	10	45	(24–68)			29	28	(19–37)		

HAM-D	Mean	SD		Mean	SD	Effect size F, partial eta^2^	P-value***		Mean	SD	Effect size Z, *r*	P-value****
Baseline	10.9	6.2		10.0	6.4	1.62, 0.02	0.21		10.7	6.2	7.43, 0.73	> 0.0001
end point	4.5	3.3		5.6	5.7				4.8	3.9		
Δ score	− 6.4	6.2		− 4.4	6.2				− 5.9	6.3		

*HAM-D* Hamilton Depression Scale, *CI* confidence interval

* Wald test statistic p-value from logistic regression model comparing Program to Control outcomes, adjusted for seizure at baseline

** McNemar’s Exact χ^2^ test for binomial paired data: comparing baseline to end point proportion of clinical outcome (i.e., recent attack) for the full cohort

*** P-value taken from ANCOVA (analysis of covariance) for each scale comparing program vs. control scores at end point, adjusted for baseline score

**** P-value from Wilcoxon Signed-Rank Test comparing baseline to end point scores (full cohort)

**Table 5:** Functioning and familial burden for epilepsy

	Program (n = 71)		Control (n = 20)		ANCOVA		Full cohort (n = 91)			
	Mean	(SD)	Mean	(SD)	Effect size F, partial eta^2^	P-val*	Mean	(SD)	Effect size Z, *r*	P-val**
*WHODAS*					0.72, < 0.01	0.40			6.38, 0.67	< 0.0001
Baseline	35.6	(20.9)	25.8	(21.3)			33.5	(21.3)		
end point	10.2	(21.1)	12.8	(25.7)			10.8	(22.1)		
Δ score	− 25.4	(28.4)	− 13.1	(21.7)			− 22.7	(27.4)		
*BAS*					< 0.01, < 0.01	0.98			8.09, 0.85	< 0.0001
Baseline	73.0	(7.9)	69.1	(9.0)			72.1	(8.3)		
end point	58.4	(6.6)	57.6	(6.4)			58.2	6.5		
Δ score	− 14.6	(9.0)	− 11.5	(8.6)			− 13.9	(8.9)		

*WHODAS* World Health Organization Disability Assessment Schedule 2.0 Short Version, *BAS* burden assessment schedule

* P-value taken from ANCOVA (analysis of covariance) for each scale comparing program vs. control scores at end point, adjusted for baseline score

** P-value from Wilcoxon Signed-Rank Test comparing baseline to end point scores (full cohort)

We found no evidence of significant differences in endpoint scores, adjusted for baseline scores, for clinical or functional outcomes across treatment arms (*P* > 0.15 for all measures), *except* the clinical outcome for epilepsy which indicated a greater reduction in recent seizure between within the comprehensive treatment arm (*P* = 0.03). Patients with epilepsy in the comprehensive arm had only 0.52 (95% CI 0.29–0.95) the risk of patients in the medication-only arm at end point after adjusting for recent seizure at baseline (*P* = 0.03). See Tables 2, 3, 4 and 5.

When reviewing for changes in functioning and familial burden for the combined psychosis and epilepsy cohorts, we found significant improvements in functional impairment (Z = 7.83, r = 0.63), familial burden (BAS: Z = 10.56, r = 0.85), and no significant differences when comparing the treatment arms. See Table 6.

**Table 6:** Functional impairment combined for epilepsy and psychosis

	Program		Control		ANCOVA		Full cohort (n = 156) T			
	Mean	(SD)	Mean	(SD)	Effect size F, partial eta^2^	P-val*	Mean	(SD)	Effect size Z, *r*	P-val**
WHODAS					1.78, 0.01	0.18			7.83, 0.63	< 0.0001
Baseline	39.9	(22.8)	31.2	(23.8)			37.8	(23.2)		
end point	15.4	(21.8)	19.4	(28.7)			16.3	(23.6)		
Δ score	− 24.5	(27.8)	− 11.8	(33.5)			− 21.5	(29.6)		
BAS					0.01, < 0.01	0.92			10.56, 0.85	< 0.0001
Baseline	74.4	(8.1)	72.2	(9.2)			73.9	(8.4)		
end point	60.4	(6.9)	59.6	(8.1)			60.2	(7.2)		
Δ score	− 14.0	(8.8)	− 12.6	(10.0)			− 13.7	(9.1)		

*WHODAS* World Health Organization Disability Assessment Schedule 2.0 Short Version, *BAS* burden assessment schedule

* P-value taken from ANCOVA (analysis of covariance) for each scale comparing program vs. control scores at end point, adjusted for baseline score

** P-value from Wilcoxon Signed-Rank Test comparing baseline to end point scores (full cohort)

## Discussion

The present study evaluates patient-level outcomes of a mhGAP-based mental health care package for people with psychosis and epilepsy in rural Nepal. The findings provide strong evidence that the cohort achieved substantial gains in every domain after 12 months of treatment, including: psychotic symptoms, seizure frequency, and depressive symptoms; functional impairment; burden for the caregiver and family; and social behavior. This means that primary health care workers, after receiving a brief training, are providing mental health care leading to significant improvements among participants. This is especially salient given the fact that the health workers in rural Nepal are all paramedics.

We also compared subgroups within the cohort, namely those that received the comprehensive MHCP consisting of multiple community and facility-level interventions versus a minimal package (eTAU) consisting only of training of health workers and supply of psychotropic medicines. The limitation of sample size inhibits our ability to make definitive interpretations of findings. Still, nearly all analyses suggest no difference in outcomes across treatment arms for all domains, with one exception: patients with epilepsy in receiving the comprehensive MHCP had improved clinical outcomes (i.e., fewer seizures) compared to those receiving the basic MHCP. We did not evaluate the impact of the added components on treatment maintenance and adherence.

This finding suggests that for treating patients with epilepsy extra investment should be made in a treatment package that includes medication *as well as* counselling, patient support groups and stigma reduction interventions. This should be further evaluated through cost-effectiveness research, in order to assess whether it is worth the additional investments. For any other outcomes, there is no additional gain for an elaborated offer of services compared to training and supply of medication only.

### Limitations

The primary limitation of our analyses is a small sample size. The final sample size after attrition and exclusion of patients with missing data was n = 179 for measures administered through clinician interview, and fewer for those administered through family interviews. This limitation is common to studies in low-resource settings; moreover, a relatively small sample size is a hallmark of cohort studies. The findings for primary objectives are not substantially affected by the sample size. It is in the comparisons of secondary objectives where the small sample size affects our ability to conduct subgroup analyses and will further limit findings should additional inferential analyses be conducted. Future research may consider testing specific hypotheses in addition to those presented here. For example, how might treatment adherence affect disorder-specific outcomes, or are beliefs regarding the cause of psychosis associated with improvements in familial burden and functional impairment? Finally, as a result of the absence of a control group that received no treatment at all (deemed unethical in the context of this study), we cannot empirically attribute change to the treatment. However, given the chronicity of the epileptic and psychotic symptoms, and the strong effects found, it is highly plausible that treatment has contributed to such change.

## Conclusions

The findings provide strong evidence for clinical improvements following mental health care delivered by primary health care workers—even paramedic staff—in treating epilepsy and psychosis. The study also shows the effect of that treatment in symptoms of depression, functional impairment and feelings of burden among family members. A more comprehensive package of care, including counselling and patient support groups, appears to offer added clinical benefits for patients with epilepsy. This entails that investments for additional psychosocial services beyond the chiefly pharmacological treatment provided by the health workers translate in significantly better treatment results. At the same time, for people with psychosis the basic package of care (i.e., psychotropic medications) performed similar to the more comprehensive package, suggesting a less resource-intensive package may offer comparable results. In conclusion, our findings demonstrate the ability of this treatment model to significantly improve the lives of those with MNS disorders and those their families. These findings also demonstrate the viable of a scalable, integrated treatment model, which, if adopted into the wider healthcare system, could dramatically expand access to mental health care for people with MNS disorder in Nepal.
